# Regulation of Intersubunit Interactions in Homotetramer of Glyceraldehyde-3-Phosphate Dehydrogenases upon Its Immobilization in Protein—Kappa-Carrageenan Gels

**DOI:** 10.3390/polym15030676

**Published:** 2023-01-29

**Authors:** Olga Makshakova, Maria Antonova, Liliya Bogdanova, Dzhigangir Faizullin, Yuriy Zuev

**Affiliations:** Kazan Institute of Biochemistry and Biophysics, FRC Kazan Scientific Center of RAS, 2/31 Lobachevsky Str., 420111 Kazan, Russia

**Keywords:** glyceraldehyde-3-phosphate dehydrogenase, κ-carrageenan, protein-polysaccharide interactions, immobilization, FTIR spectroscopy, molecular docking

## Abstract

Polysaccharides, being biocompatible and biodegradable polymers, are highly attractive as materials for protein delivery systems. However, protein–polysaccharide interactions may lead to protein structural transformation. In the current study, we analyze the structural adjustment of a homotetrameric protein, glyceraldehyde-3-phosphate dehydrogenase (GAPDH), upon its interactions with both flexible coil chain and the rigid helix of κ-carrageenan. FTIR spectroscopy was used to probe the secondary structures of both protein and polysaccharide. Electrostatically driven protein–polysaccharide interactions in dilute solutions resulted in an insoluble complex formation with a constant κ-carrageenan/GAPDH ratio of 0.2, which amounts to 75 disaccharide units per mole of protein tetramer. Upon interactions with both coiled and helical polysaccharides, a weakening of the intersubunit interactions was revealed and attributed to a partial GAPDH tetramer dissociation. In turn, protein distorted the helical conformation of κ-carrageenan when co-gelled. Molecular modeling showed the energy favorable interactions between κ-carrageenan and GAPDH at different levels of oligomerization. κ-Carrageenan binds in the region of the NAD-binding groove and the S-loop in OR contact, which may stabilize the OP dimers. The obtained results highlight the mutual conformational adjustment of oligomeric GAPDH and κ-carrageenan upon interaction and the stabilization of GAPDH’s dissociated forms upon immobilization in polysaccharide gels.

## 1. Introduction

Glyceraldehyde-3-phosphate dehydrogenase (GAPDH, EC 1.2.1.12) is a housekeeping protein associated with both human health and diseases [1,2]. GAPDH is mostly known as an energy-metabolism-related enzyme. As one of the key enzymes of glycolysis, it catalyzes the oxidative phosphorylation of glyceraldehyde-3-phosphate to 1,3-diphosphoglycerate, coupled with the reduction of the cofactor NAD+ to NAD(H). The protein is also involved in a wide range of cellular functions [3,4], including the induction of apoptosis [5] and the development of neurodegenerative diseases, e.g., Alzheimer’s disease and Parkinson’s disease [6]. 

**Figure 1:**
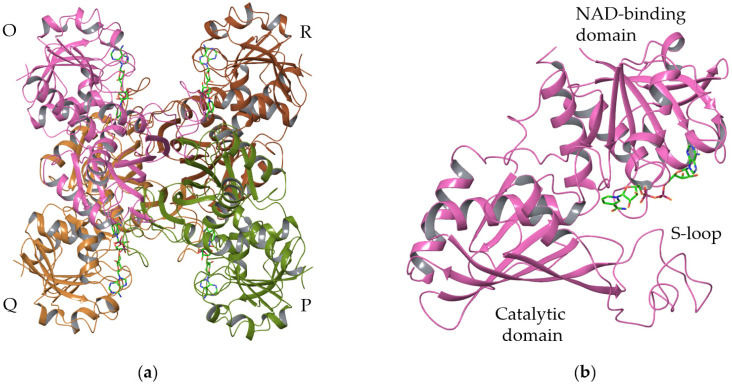
GAPDH oligomeric (**a**) and domain structure (**b**).

GAPDH is a tetrameric protein consisting of four identical subunits. It has three axes with double symmetry (Figure 1) and can be represented as a dimer of dimers with respect to the Q axis. Each of its subunits consists of two domains, catalytic (aa 151–316) and NAD-binding (aa 1–150 and 317–335). The NAD-binding groove is located between the O- and R- (P- and Q-) subunits, which interact mainly through the S-loop. Such oligomeric organization stabilizes the protein and regulates the GAPDH activity. The strengthening of intersubunit interactions was shown to provide enhanced thermostability [7]. In contrast, the destabilization of intersubunit interactions and the dissociation of the GAPDH tetramer into smaller units is important for the processes related to transcytosis and the modulation of aggregation of other proteins [8,9].

For example, GAPDH is translocated to the nucleus during apoptosis [10] in non-native dimeric, monomeric, and denatured forms [11]. Modifications, such as oxidation, may assist with the dissociation of the tetramer into subunits, which move to the nucleus due to passive transport [12]. Other factors affecting the GAPDH tetramer dissociation under physiological conditions are interactions with either negatively charged membranes [13,14] or the polysaccharides of the extracellular matrix [15]. A similar effect was described upon denaturing conditions: in the presence of guanidine hydrochloride (GdnHCl), the enzyme also first dissociated into dimers or monomers and then aggregated and precipitated. In the presence of small quantities of GdnHCl, the dissociated GAPDH intermediate was stable for hours [16]. However, in the presence of charged surfaces, the dissociation of GAPDH was followed by the accumulation of amyloid-like β-sheet conformation [13,15]. 

Several studies demonstrated that GAPDH is colocated with amyloid beta (Aβ) protein in the amyloid plaques [17,18], where it is colocalized with glycosaminoglycans (GAGs) [9]. The role of the sulfated polysaccharides of the extracellular matrix in amyloid formation is well-documented [19]. However, it is still unclear to what extent the type of polysaccharide chain and the pattern of sulfation destabilize the protein structure. Heparan sulfate (HS) was capable of inducing GAPDH aggregation, but chondroitin sulphates (CS) A, B and C and dextran sulphate revealed almost no effect [15]. It is assumed that the position of the sulfate groups on the polymeric sugar chain is more important than their density regarding GAPDH aggregation [15].

In the current study, we analyze how κ-carrageenan, a sulfated seaweed polysaccharide, influences the GAPDH conformation upon its complexation in gels. A major emphasis is placed on the effect of the polysaccharide’s secondary structure on the GAPDH structure’s adjustment. Carrageenans are linear-sulfated galactans from red seaweeds (*Rhodophyta*). Their molecules consist of galactose repeat units, where one ring per disaccharide can be (3,6)-anhydro, connected by alternating α-(1,3)- and β-(1,4)-glycosidic links. One to three sulfate groups can be attached to a carrageenan chain per monomeric unit. Similar to other sulfated polysaccharides [20], carrageenans can be considered as GAG mimetics, revealing their potential for drug delivery and tissue engineering [21,22,23]. κ-Carrageenan demonstrates a controllable coil-to-helix transition upon gelation depending on the salt type and content and temperature variations. It is generally accepted that κ-carrageenan chains associate into a parallel inter-twisted double helix [24]. This double helix was deduced from the refinement of the X-ray pattern of the oriented fibers of its near analogue, ι-carrageenan [25]. As derived from theoretical calculations, the spatial motif of a κ-carrageenan chain is a threefold right-handed helix [26]. At low concentrations or upon heating, the polysaccharide chain acquires flexibility and adopts a random coil conformation. Therefore, along with variations in the sulfation pattern, carrageenans offer a way to vary chain conformation/rigidity and the accessibility of the sulfate groups. Recently, using multivariate analysis, we reported the clearly distinct orthogonal FTIR spectra of the helix and coil conformations of κ-carrageenan [27], which allows for probing the polysaccharide conformation in situ.

The electrostatically driven interactions between protein and negatively charged polysaccharides result in a coacervation and the formation of macromolecular complexes [28,29,30]. When the polysaccharide is of a high molecular weight, such interactions lead to phase separation and the formation of gels enriched with both protein and polysaccharides. Recently, we demonstrated that coiled carrageenan being mixed with lysozyme forms gels at a stoichiometry 1:3 mass ratio of polysaccharide to protein (or 12 disaccharide units per protein molecule). In such gels, the carrageenan remained a coil, but the protein adjusted its conformation toward an increase in the intramolecular β-structure, as revealed by FTIR spectroscopy. Strikingly, the helical conformation of the polysaccharide did not influence the protein structure so drastically [31]. In the present study, we address the question of structural changes in GAPDH—κ-carrageenan gels by using FTIR spectroscopy to simultaneously follow the structural changes of both the protein and polysaccharides. This is possible, since most of the informative bands of the protein and polysaccharides do not overlap. Further, to provide an atomistic model of complexes, molecular docking was applied.

The results of the current research expand our knowledge about the interaction-induced structural transformations that occur upon protein immobilization in polysaccharide matrixes and highlight the role of the polysaccharide’s secondary structure and its rigidity, together with the charge distribution in controllable structural transitions.

## 2. Materials and Methods

### 2.1. Materials and Sample Preparation

GAPDH (G2267) and κ-carrageenan (BCBT9953, 2204-25G-F) were purchased from Sigma-Aldrich. GAPDH was dialyzed against milliQ water for 24 h to wash out the excess of salt. κ-carrageenan was used without additional purification. The viscosity average molecular weight amounted to Mη = 4.3·10^5^ Da, as reported previously [32].

Aqueous solution of κ-carrageenan was allowed to swell for one hour at 20 °C and then dissolved by steering for two hours at 70 °C. The pH of κ-carrageenan solution amounted to 9.0 and was brought to 7.1 by HCl addition. The stock aqueous solutions of κ-carrageenan and GAPDH (pH 7.1) were mixed at 25 °C to obtain the desired polysaccharide/protein ratio (q) between 0.1 and 1, with a step of 0.1. The portions of stock solutions of GAPDH (20 mg/mL) and κ-carrageenan (5,5 mg/mL) were mixed, and then the volume was brought to 500 mkL by adding milliQ water. The concentration of protein in the resultant mixtures was kept constant at 5 mg/mL.

To have mixtures of GAPDH with κ-carrageenan prone to form helical structure, more concentrated κ-carrageenan solution was prepared (40 mg/mL) as described above. The GAPDH solution was added to two vials containing the polysaccharide, either cool or heated up to 45 °C (temperature of helix-to-coil transition). The samples were kept for 4 h before spectra registration.

The amount of protein in solution or in water soluble fraction above gel after centrifugation (supernatant) was calculated from the intensity of UV spectra at 280 nm using ε_GAPDH_ = 30.4 mM^−1^cm^−1^ [33].

### 2.2. ATR-FTIR Spectroscopy

The FTIR spectra were recorded using IRAffinity-I spectrometer (Shimadzu, Europa GmbH) equipped with the attenuated total reflection (ATR) accessory and a ZnSe crystal. The spectra were recorded at 4 cm^−1^ resolution, in the range of 4000–800 cm^−1^. For each spectrum, 256 scans were accumulated. The samples of solutions and gels were placed on the surface of ATR sensing element and were thermostated at 25 °C. The spectra of the samples, including stock solutions of GAPDH and κ-carrageenan, were also recorded. From all spectra, both water vapor and liquid water absorbance were subtracted.

### 2.3. Molecular Docking

The coordinates from protein data bank (1ZNQ) were used to construct four types of GAPDH assembly in apo form (as in Ref. [34]): 1. tetramer, 2. OP dimer, 3. OR dimer, and 4. monomer. The coordinates of ι-carrageenan (1CAR) were used to construct the initial geometry of both single and double chain of κ-carrageenan (as in Ref. [32]). The sulfate group of ι-carrageenan at O2 position of anhydrogalactose unit was replaced with a hydroxyl group. The obtained structures were energy-minimized before the docking procedure. To mimic a flexible chain, the κ-carrageenan was reduced to a hexamer fragment to avoid the conformational sampling problem in such a highly flexible polymer. The efficiency of fragment-based docking approach for the polysaccharide binding mode search was demonstrated previously [35,36]. During docking, all torsions were flexible to mimic a flexible chain in a coil conformation. The resultant docking poses were checked for the allowed values of glycoside torsion angles (the phi-psi maps are available in Supplemental Materials to Ref. [32]). In double helix, only hydroxyl groups were allowed to rotate to maximize the hydrogen bonds with the protein.

In the blind docking using AutoDock4.2 program [37], the grid covered the entire protein molecule, so the ligand could bind to any part of the protein surface. To navigate the orientation of polar groups in the protein binding site, the solvent-biased docking approach was applied. This approach takes into consideration the solvent structure (calculated in the course of molecular dynamics simulations) and demonstrated its efficiency to improve the prediction of both specific carbohydrate binding (e.g., by lectins [38]) and non-specific polysaccharide binding (e.g., by RNAse [35]). Further, the grid map was modified using WATCLUST [39], which calculates the water clusters over the protein surface based on the 20 ns molecular dynamics trajectories of each of four types of GAPDH assembly in water and introduces this information for the particular interaction of ligand oxygen atoms. For consistency, the protocol of molecular dynamics calculation is described below. In the docking runs, the protein molecule was kept rigid, whereas in ligand, it was fully relaxed. Lamarckian genetic algorithm was used for the sampling. For each protein system, 100 independent docking runs were carried out, and 1000 best positions were obtained for the analysis. The docking poses were ranked using the binding energy, which included electrostatic and van der Waals interactions, hydrogen bonding, solvation effects and conformational entropy. The cluster analysis was performed with the positional rmsd cut-off of 15 Å, to obtain the population at a local spot, irrespective of the orientation of the polysaccharide fragment. The plot of population (i.e., the number of docking poses in one cluster) versus binding energy was used to distinguish between true positives and false positives.

To compare different systems, the binding energies were recalculated per disaccharide, dividing the value by a consistent number of dimers directly interacting with the protein (e.g., 3 for hexameric fragment and 10 for the double helical fragment of κ-carrageenan).

The molecular dynamics simulations were performed in AMBER12SB force field using AMBER12 program package [40]. Each protein system was immersed into TIP3P water box with periodical boundary conditions. The edges of box were 10 Å apart from the solute molecule. Atoms of Cl- were used to neutralize the total charge of molecular system. Particle mesh Ewald (PME) [41] was used for the long-range electrostatic interactions, with cut-off of 8 Å. Temperature and pressure were kept constant using the Langevin thermostat, with a collision frequency of 2 ps^−1^. Integration time of step 2 fs was used with the SHAKE algorithm [42]. The system was first energy-minimized and then equilibrated in NPT heated to 300 K, and the pressure was kept equal to 1 bar. In production run, the trajectory of 20 ns long was accumulated.

## 3. Results and Discussion

### 3.1. FTIR Spectroscopy Analysis

#### 3.1.1. Gels of GAPDH and κ-carrageenan in Coil Conformation

Mixing GAPDH and κ-carrageenan solutions resulted in a simultaneous insoluble complex formation. Within the panel of mixtures that were varied by the polysaccharide—protein mass ratio, from 0.1 to 1 by an increment of 0.1, a stoichiometric amount of GAPDH and κ-carrageenan precipitated in the form of gels. Stoichiometry was achieved at q = 0.2. Below this ratio, the protein concentration in precipitate increased and then was retained at the same amount. The protein concentration in the sediment was calculated as the difference between the initial protein amount and the protein amount in the supernatant, which was estimated using UV spectrophotometry. This observation is in line with the conclusion from the FTIR spectra, where an excess of protein, without κ-carrageenan bands, appeared in the spectra of the supernatant at values below q = 0.2. Above this value, only bands of κ-carrageenan were visible in the FTIR spectra of the supernatant. A similar behavior was described for the mixtures of lysozyme and κ-carrageenan [31]. The stoichiometry can be explained by the charge compensation between protein and κ-carrageenan [28,30]. In these complexes, the ratio amounts to 75 disaccharide units per GAPDH tetramer molecule. Note that at the used concentrations and conditions κ-carrageenan itself does not gel, but it can gel in the presence of proteins. Our results indicate that GAPDH may cross-link the flexible κ-carrageenan chains, similar to lysozyme [31].

**Figure 2:**
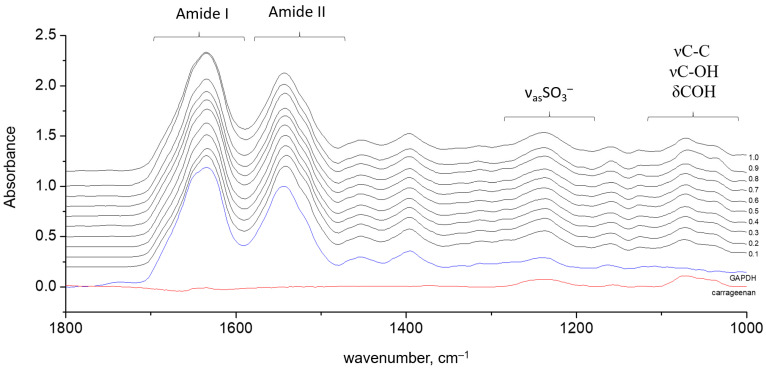
Spectra of κ-carrageenan—GAPDH gels obtained by mixing at different mass ratios of 0.1, 0.2, 0.3, 0.4, 0.5, 0.6, 0.7, 0.8, 0.9 and 1.0. The reference spectra of pure GAPDH (blue) and κ-carrageenan in coil conformation (red) are given for comparison.

The gel spectra revealed the constant ratio of the protein and polysaccharides in the sediments (Figure 2). Two intense bands, Amide I and Amide II, are distinctive protein vibration bands. The Amide I band results from the peptide C=O stretching vibrations. The Amide II band results from a combination of N–H bending and C–N stretching vibrations. The absorbance of polysaccharides appears below 1300 cm^−1^. The complex band in the region 1200–1300 cm^−1^ is attributed to the sulfate groups stretching. The absorbance at 1200–1000 cm^−1^ is called the fingerprint region, due to a high number of overlapping bands. The bands arise from the collective vibrations of pyranose rings νC–C, νC–OH and δCOH [43,44,45]. Despite the precise assignment of band components being difficult, a correlation was found between the relative intensities of the components in the range 1000–1100 cm^−1^ and the conformation of the polysaccharide chain [27,46]. The protein absorbance in this range is weak and could be readily subtracted. The contour shape of the polysaccharide’s absorbance bands in the range of 1000–1100 cm^−1^ undoubtedly allows for attributing it to the polysaccharide’s coil state.

As seen from Figure 2, the protein bands that are sensitive to peptide conformation, Amide I and II, are free from carrageenan absorbance. The only interference comes from the δOH of residual water at 1640 cm^−1^, which appears as a broad incurvature of the baseline but does not contribute to the second derivative spectra. The fact that the conformation-sensitive bands of the GAPDH and κ-carrageenan do not overlap allows us to simultaneously analyze the structural rearrangements of both the protein and polysaccharide upon complex formation.

**Figure 3:**
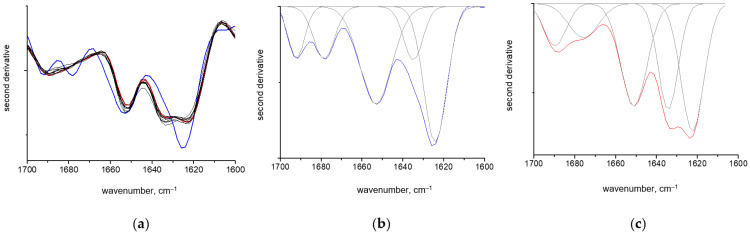
Second derivative spectra of GAPDH solution (blue) and GAPDH–κ-carrageenan mixtures at different mass ratios of 0.1, 0.2, 0.3, 0.4, 0.5, 0.6, 0.7, 0.8, 0.9 and 1.0 in Amide 1 region (**a**). Fitting of second derivative spectra of GAPDH solution (**b**) and the mixture at the ratio 0.2 (**c**) with Gaussian peaks in Amide 1 region.

Figure 3a shows the second derivative spectra in the Amide I region. The band consists of a series of components resulting from the different elements of the protein’s secondary structure [47,48]. For native GAPDH, the component at 1655 cm^−1^ was assigned to a α-helix, and the components at 1624 and 1639 cm^−1^ were assigned to a β-structure [16,47]. The high frequency bands at 1674–1696 cm^−1^ are due to the various types of β-turns. In addition, the absorbance of side groups appears around 1610–1630 cm^−1^ [49]. Upon binding to κ-carrageenan, the second derivative spectra of GAPDH revealed a change in the spectral contour shape. The intensity at 1624 cm^−1^ decreased, while the intensity at 1639 cm^−1^ increased. The intensity at 1655 cm^−1^ remained the same, as in the GAPDH solution. The decrease in the intensity at 1624 cm^−1^ was reported during GAPDH treatment with 0.1 M GdnHCl and was attributed to the tetramer dissociation. The following aggregation was associated with the appearance of a sharp band at 1620 cm^−1^. It should be noted that, in contrast to GdnHCl denaturation or amyloid formation in the presence of lipid membranes [13] or heparin [15], no further steps of GAPDH denaturation were detectable in the gels with κ-carrageenan, and only partial tetramer dissociation was detected.

Fitting the second derivative spectrum [47] of native GAPDH with five Gaussian components (Figure 3b) revealed the presence of 45% β-sheets, 34% α-helices and 21% β-turns. These values are close to those from the X-ray structure, which were 40% β-sheets and 36% α-helices, respectively [50]. Minor variations from the X-ray data are possible when FTIR spectroscopy is used for such estimation (around an 8% mismatch was reported in [16]). Fitting the second derivative spectrum of GAPDH in gels with κ-carrageenan revealed a change in the area, mainly under two Gaussian components, when compared to the pure GAPDH spectrum. The decrease in the component at 1624 cm^−1^ was compensated for by the increase in the component at 1635 cm^−1^ (Figure 3c). The content of the α-helices remained around 30%. A minor variation in the area of the component at 1655 cm^−1^ was due a slight band narrowing in the gel spectra. To avoid arbitrariness, when possible, the bandwidth was kept constant in the course of contour fitting. Previously, it was reported [16] that upon denaturation the band attributed to the helical structure at 1655 cm^−1^ did not change. In addition, the authors claimed the only band at 1624 cm^−1^ disappeared. However, provided that the area of the whole Amide I band is 100%, diminishing the band at 1624 cm^−1^ occurs at the expense of the band at 1635 cm^−1^, which becomes more intense. A similar rise of the β-sheets absorbance upon interactions with κ-carrageen was observed in lysozyme [31].

#### 3.1.2. Gels of GAPDH and κ-carrageenan in Helical Conformation

**Figure 4:**
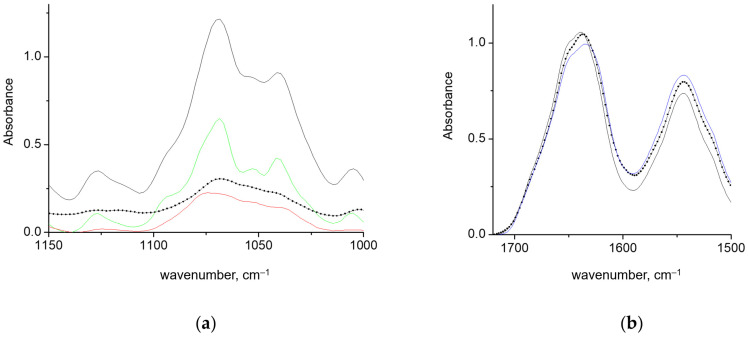
The absorbance spectra of κ-carrageenan in the conformation of helix (green) and coil (red) and the spectra of a GAPDH–κ-carrageenan gels at mixing at 25 °C (dotted black) and 40 °C (solid black) in the region of polysaccharide absorbance (**a**). The spectra of GAPDH solution (blue) and the spectra of a GAPDH–κ-carrageenan gels at mixing at 25 °C (dotted black) and 40 °C (solid black) in the region of Amide I and Amide II (**b**).

To further explore the influence of a rigid helical polysaccharide conformation on the GAPDH intersubunit interactions, the experiment was performed with higher concentrations. At the conditions and concentrations used, κ-carrageenan forms elastic gels that melt, starting at ~40 °C [27]. Upon admixing the protein solution to κ-carrageenan gel at room temperature, the polysaccharide retained a helical conformation with sharp bands in the 1000–1100 cm^−1^ region, as evidenced by the FTIR spectra (Figure 4a). In contrast, when GAPDH solution was added to κ-carrageenan at 45 °C and then cooled down, the spectral contour of κ-carrageenan was rather smoother, and the components were less distinguishable. Such a contour shape remained a rather typical one for coil conformation (Figure 4b). However, the second derivative spectra showed that the components are located at the same position as in the spectrum of helical κ-carrageenan. The broadening of the components indicated a decrease in the helix quality. This implies that the interactions of a carrageenan chain with GAPDH perturbed the κ-carrageenan’s interchain interactions. This behavior differed from when a small globular protein, lysozyme, was mixed with a warm κ-carrageenan solution. In the latter case, κ-carrageenan formed a helical structure and gelled upon cooling down, which extracted an excess of protein. Despite some amount of lysozyme being entrapped into the gel, the spectral features of polysaccharide were the same as those in pure κ-carrageenan gel [31]. In contrast, the unwinding of the carrageenan double helix was reported to be induced by β-casein [51].

The Amide I in the GAPDH spectra is characterized by a reduced absorbance at 1624 cm^−1^ and a dominant band at 1635 cm^−1^, for both types of mixing with concentrated κ-carrageenan. Other bands overcome only minor changes. Such spectral deviations are due to direct interactions of GAPDH with κ-carrageenan and should not be attributed to temperature effects. Differential scanning calorimetry (DSC) showed that GAPDH structure was stable below 45 °C. The first DSC peak attributed to the intersubunit dissociation spanned 45–70 °C, with a maximum at 58 °C [7].

Thus, GAPDH tends to interact with κ-carrageenan as a partially dissociated tetramer (into dimers or monomers) rather than as a whole tetramer. Such interactions are favorable upon interactions with both the coil and helical chains of κ-carrageenan.

### 3.2. Computer Modeling of Interactions between GAPDH in Different Oligomeric States and κ-carrageenan

Based on our experimental data, we hypothesize that the distortion of the GAPDH tetrameric structure in κ-carrageenan gels can result from the stabilization of the protein dimers by direct interactions with the polysaccharide chains. To estimate which interactions of κ-carrageenan with GAPDH can prevent tetramer assembly, the question was further addressed by computer modeling. To analyze the molecular basis of these interactions, we performed molecular docking on four types of receptor molecules: 1. GAPDH tetramer, 2. OP-dimer, 3. OR-dimer, and 4. monomer. The OQ type of dimer was not taken into consideration, since it is the weakest type of GAPDH dimer and is least probable in solution [7].

**Figure 5:**
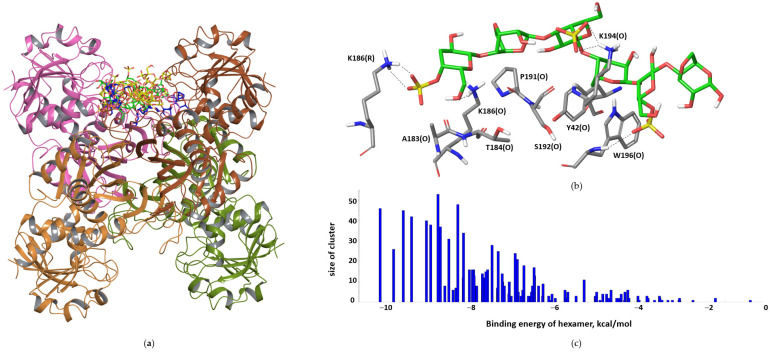
The superimposition of the most favorable binding poses located in the OR groove of tetramer (carbon atoms in yellow), OR-dimer (carbon atoms in green) and OP-dimer (carbon atoms in blue) (**a**). A representative of the binding mode to GAPDH tetramer (**b**). The plot of cluster size versus its binding energy (**c**).

All four receptors shared a highly populated binding site located in the region of the NAD-binding sites, as illustrated on the structure of the tetramer in Figure 5. Over all poses, this one was the most favorable, gaining −3.3 kcal/mol per disaccharide unit. The interactions mainly involve the polar and basic residues of the S-loop: T184, K186, P191, S192 and K194.

**Figure 6:**
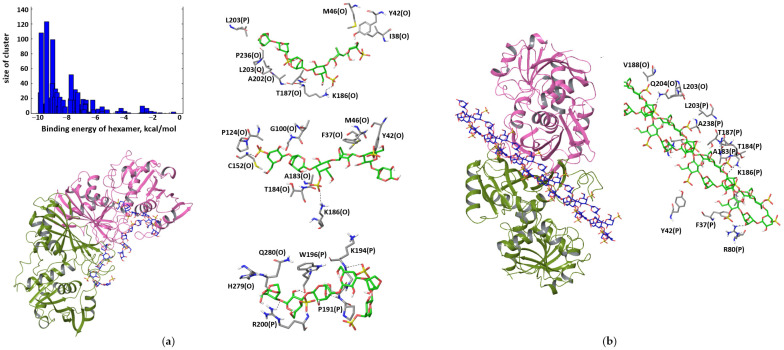
Docking poses of flexible κ-carrageenan chain in OP-dimer that may prevent tetramer formation (**a**). The most favorable binding pose of κ-carrageenan double helix in the OP dimer (**b**).

In the OP-dimer, two other epitopes are occupied with true positive docking poses. They comprise the helix L40–Y49, namely aromatic and non-polar residues F37, Y42 and M46, and parts of the S-loop: P191, K194, W196 and R200. Three docking hits illustrate the favorable modes of interactions with these two epitopes (Figure 6a). In tetramers, these two epitopes come into direct contact with each other, stabilizing the interactions of the two dimers of the OP type. The binding energy is −3.0 kcal/mol per disaccharide. When GAPDH is exposed to κ-carrageenan, the protein–polysaccharide interactions may screen the OP dimer from protein–protein interactions, thus stabilizing the dimer state. The binding of the double helix of κ-carrageenan occurs in the region of the NAD-binding site and crosses the inner part of the OP dimer (Figure 6b), which is buried in the tetramer. The binding of the rigid helix is less favorable compared to the flexible chain and amounts to 0.9 kcal/mol per disaccharide unit.

**Figure 7:**
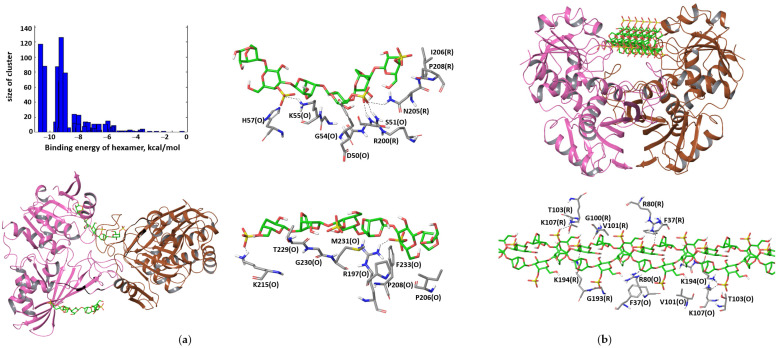
Docking poses of flexible κ-carrageenan chain in OR-dimer that may prevent tetramer formation (**a**). The most favorable binding pose of κ-carrageenan double helix in the OR dimer (**b**).

In the OR-dimer, the binding poses that may interfere with the tetramer formation are located at β-strands N239–G247 and A232–P236, which are located at the edge of the large β-sheet involved in the intersubunit interactions in the OP-dimer. The closest contacts are formed with residues N205, I206, P208 and F233 (Figure 7a). The complexes are stabilized by the interactions with the basic residues of the S-loop, namely, R197 and R200 (Figure 7a). The binding energy is −3.1 kcal/mol per disaccharide unit. Despite κ-carrageenan offering both hydrophobic and hydrophilic patches to interact with protein and probably interacting with both the basic residues of the S-loop and the hydrophobic residues of the OP contacts, the destabilization of the OP dimer seems to be less probable due to the vast hydrophobic interactions across the OP intersubunit’s contact. However, the quality of these contacts can be distorted due to the favorable interactions with the polysaccharide The double helix of the carrageenan binds to the groove (Figure 7b), with an energy of −1.4 kcal/mol per disaccharide unit.

In agreement with our results, positively charged grooves, located between the O and R (P and Q) subunits and spanning ~70 Å, were proposed as a probable binding site for other negatively charged biopolymers, including heparin [15], DNA and RNA [52,53]. However, the results of the available modeling were obtained on the GAPDH tetramer. Dealing with the constituent parts of the tetramer allows us to reveal new binding sites in the intersubunit contacts, as exemplified by κ-carrageenan. The competitive interactions with polymers in intersubunit contacts may stabilize GAPDH in a particular oligomeric state. The understanding of the factors that bias the equilibrium of the protein assembly and regulate the enzyme properties upon their immobilization is crucial for biotechnological tasks and creates a platform for the design of “smart polymers” [54,55].

## 4. Conclusions

In the present article, we report a study on the conformational alternations in a GAPDH tetramer upon immobilization in κ-carrageenan gels. The conformation of the stock polysaccharides varied from a coil to a rigid helix. The conformational adjustment of the interacting protein and polysaccharide was controlled using FTIR spectroscopy. The complex formation was accompanied with changes in the protein spectra that were assigned to the GAPDH tetramer dissociation. This dissociation was observed in both cases, when the polysaccharides were in coil and helix conformations. This implies that κ-carrageenan interacts with non-native forms of GAPDH more favorably than with its native tetrameric form. To test the hypothesis that direct interactions with the polysaccharide may shift the association–dissociation equilibrium in GAPDH, molecular modeling of different GAPDH oligomers was carried out. Molecular docking revealed that κ-carrageenan favorably binds to the epitopes that form protein–protein contacts in the tetramer. The most interfering interactions were found in the contacts between the OP and QR dimers. Therefore, it was concluded that, in κ-carrageenan gels, GAPDH is stabilized as an OP (OR) dimer. Furthermore, the results of our study demonstrate that the incorporation of GAPDH perturbs the κ-carrageenan gel network, degrading the quality of the polysaccharide helices. The obtained results highlight the mutual conformational adjustment of oligomeric GAPDH and κ-carrageenan upon interaction and the stabilization of GAPDH’s dissociated forms upon immobilization in the polysaccharide gels.

## Data Availability

The data in this study are available on reasonable request from the corresponding author.
